# Book Reviews

**Published:** 1917-12

**Authors:** 


### BOOK REVIEWS

Books mentioned may be inspected at and ordered through this office. So far as possible, books received in any month will be reviewed in the issue of the second month following. Pamphlets, quarterly and similar periodicals, reports, transactions, etc., will, as a rule, merely be mentioned.

**Reference Hand Book of the Medical Sciences.** Vol. 8, finishing the alphabet and including an appendix and index, has appeared, completing the third revised edition begun in 1913.

The publishers, Wm. Wood & Co. and the editor, Dr. Thomas Lathrop Stedman, are to be congratulated, first of all on the mere fact of having completed this monumental task. The mere bigness of it impresses one so much that considerations of quality take second place. Imagine the responsibility for some eight million words, and four thousand illustrations. Of course, the work has been divided among many authors but even so,—and we speak from definite knowledge on this point—it has been planned in detail in advance, so that all phases of medicine might be covered in the alphabetic arrangement, and critically edited. The success of the work is due to elaborate attention to details under a comprehensive plan. Omissions have been reduced to a negligible factor by years of checking the previous two editions, the first of which appeared about 30 years ago.

**Medical Clinics of North America.** W. B. Saunders Co., Philadelphia, bi-monthly, $10 per year. Sept. 1917, Philadelphia number; Nov. 1917, N. Y. City number.

**The Rockefeller Foundation,** Annual Report for 1916. Published by the Foundation, 61 Broadway, N. Y.

This deals mainly with the work of the International Health Board which has investigated various problems in a wide range of places; of the China Medical Board; and of the War Relief Commission. The Foundation has been most generously endowed with a capital of a little over 100 million, yielding close to 6%, which has been distributed from year to year in very different amounts to a wide range of philanthropies. To grasp these large sums it is necessary to resort to simple comparisons. There are approximately 4 million automobiles in the country. Each one of these would have to contribute $1.25-$1.50 to make up the vast sum dispensed in a year for benevolences. If we imagine each of them to consume 500 gallons of gasoline a year, and that the government had raised this money by taxation, it would have had to increase the price a quarter of a cent a gallon to the consumer, beyond that taken as the normal standard of value, as established by the current rates for 1914 and 1915. Fortunately the government did not impose this tax.

---

**The Practical Medicine Series.** Charles L. Mix, A. M., M. D., general editor. The Year Book Publishers, 327 S. La Salle St., Chicago. 10 volumes in annual series, $10.

Vol. 6, General Medicine, Frank Billings, M. S., M. D., and Burrell O. Raulston, A. B., M. D., editors, 347 pages $1.50 separately.

This review of current literature has been carried on for over 10 years, in a highly satisfactory manner. The division of the literature into sections, medicine and surgery usually occupying 2 volumes each, and the grouping of smaller sections with others, offers a convenient and economic choice to those not desiring to cover the whole field of medicine and also facilitates the assembling of the notes. The excerpts are sufficiently full of references as they stand, without requiring consultation of the original sources, unless for special purposes and illustrations are frequently reproduced.

